# Evidence for neurogenesis in the medial cortex of the leopard gecko, *Eublepharis macularius*

**DOI:** 10.1038/s41598-018-27880-6

**Published:** 2018-06-25

**Authors:** Rebecca P. McDonald, Matthew K. Vickaryous

**Affiliations:** Department of Biomedical Sciences, Ontario Veterinary College, University of Guelph, Guelph, Ontario Canada

## Abstract

Although lizards are often described as having robust neurogenic abilities, only a handful of the more than 6300 species have been explored. Here, we provide the first evidence of homeostatic neurogenesis in the leopard gecko (*Eublepharis macularius*). We focused our study on the medial cortex, homologue of the mammalian hippocampal formation. Using immunostaining, we identified proliferating pools of neural stem/progenitor cells within the sulcus septomedialis, the pseudostratified ventricular zone adjacent to the medial cortex. Consistent with their identification as radial glia, these cells expressed SOX2, glial fibrillary acidic protein, and Vimentin, and demonstrated a radial morphology. Using a 5-bromo-2′-deoxyuridine cell tracking strategy, we determined that neuroblast migration from the ventricular zone to the medial cortex takes ~30-days, and that newly generated neuronal cells survived for at least 140-days. We also found that cell proliferation within the medial cortex was not significantly altered following rupture of the tail spinal cord (as a result of the naturally evolved process of caudal autotomy). We conclude that the sulcus septomedialis of the leopard gecko demonstrates all the hallmarks of a neurogenic niche.

## Introduction

Naturally evolved examples of constitutive postnatal neurogenesis have been reported for representative members of most major vertebrate (and even some invertebrate) lineages^1,2^. Although the ability to generate new neurons throughout adulthood appears to be widely conserved, there are striking differences across species^2,3^. In mammals, neural stem/progenitor cells (NSPCs) capable of continuously generating new neurons are restricted to two cardinal regions of the brain: the subventricular zone of the lateral ventricle and the subgranular zone of the hippocampus (although the latter may be absent from whales^4^). In contrast, among many non-mammalian vertebrates, including some teleost fish, salamanders, frogs and reptiles, the capacity for neurogenesis is more pervasive and includes multiple regions along the entire neuraxis^5–8^. In some teleosts, there are as many as 16 distinct neurogenic regions^9–13^. Similarly, multiple neurogenic regions of the brain have also been reported for lizards, including various locations throughout the telencephalon, as well as within the cerebellum^14–17^. The most widely studied neurogenic region is the portion of the brain homologous with the mammalian hippocampal formation, the medial cortex^15,18–20^.

In lizards (as for most vertebrates), the cell population lining the walls of the ventricular system is known as the ventricular zone^2,14,16,17^. Within the cerebral cortex, the ventricular zone includes pools of mitotic cells, predominantly within pseudostratified regions known as ependymal sulci. In reptiles, there are four ependymal sulci: the sulcus lateralis, sulcus ventralis, sulcus terminalis, and sulcus septomedialis^21^. Proliferating cells within the ventricular zone are typically identified as radial glia, NSPCs with a distinctive morphology (including a lengthy basal or radial process that spans the parenchyma of the brain to reach the pial surface of the brain, or one (or more) blood vessels) and pattern of protein expression otherwise characteristic of neuroprogenitor cells (e.g., SOX2) or astrocytes (glial fibrillary acidic protein (GFAP))^22–25^. Although evidence of cell proliferation and migration has only been reported for a small number of lizard species^14^, the accepted model is that radial glia within the sulci undergo asymmetric cell division, giving rise to immature neurons (neuroblasts), which then migrate and ultimately differentiate within the neuron-rich areas of the cortex^16,26,27^.

Although the process of neurogenesis appears to be broadly similar across lizard taxa, there are notable differences between species. For example, in the Iberian wall lizard (*Podarcis hispanicus*) migration begins 2–4 days following new cell generation (indicated by movement away from the ventricular zone) and is complete by 7 days (indicated by cells reaching the medial cortex)^18^. In comparison, cell migration takes 7 days to begin in both Moorish geckos (*Tarantola mauritanica*) and Peters’ lava lizards (*Tropidurus hispidus*), and approximately 30 days to complete^15,16^. Species-specific differences also exist in the timeframe for neuronal maturation. Newly generated cells in *T*. *mauritanica* display ultrastructural features of mature neurons within 30 days (when they reach the medial cortex)^16^. However, in Gallot’s lizards (*Galloti gallotia*) cells that have migrated to the medial cortex within 30 days still have ultrastructural features of neuroblasts (i.e., immature neurons), and do not take on characteristics of mature neurons until 90 days after their generation^26^. These findings provide intriguing evidence for unequal neurogenic potential across lizard species. It is also worth noting that, where reported, studies to date involve wild-caught populations and thus rely on estimates of lizard ages, an important factor known to influence neurogenic capacity^27,28^.

Here, we conduct an in-depth characterization of homeostatic neurogenesis in the medial cortex of the leopard gecko (*Eublepharis macularius*). Leopard geckos offer several distinct advantages for the study of lizard neurogenesis. First, leopard geckos are captive bred and commercially available. Therefore, our investigation employed a cohort of 18 near-identical aged individuals, purchased as hatchlings. All geckos were maintained under identical conditions in our facility for 146 days (see Materials and Methods) prior to the start of the experiment. At the start of the experiment this cohort had a similar mass (range of 13.0–15.9 g) and body length (snout-to-vent length, 85–100 mm). Second, leopard geckos have a well-documented capacity to regenerate multiple tissue types, including the spinal cord, following tail loss^29–33^. Tail loss (caudal autotomy) is a naturally evolved anti-predation strategy common to many species of lizards^34^. Detachment of the tail provokes a proliferative and reparative response from the ruptured end of the spinal cord, leading to spinal cord regeneration^30,35^. Previous work on rodents has revealed that the hippocampus is sensitive to spinal cord injuries^36^. Taking advantage of caudal autotomy, we sought to determine if cell proliferation in the medial cortex was altered in response to rupture of the tail spinal cord. Our findings reveal that NSPCs are present within the sulcus septomedialis of leopard geckos, and that these cells serve as the source of new neurons for the medial cortex.

## Results

### Organization of the sulcus septomedialis and medial cortex

**Figure 1:**
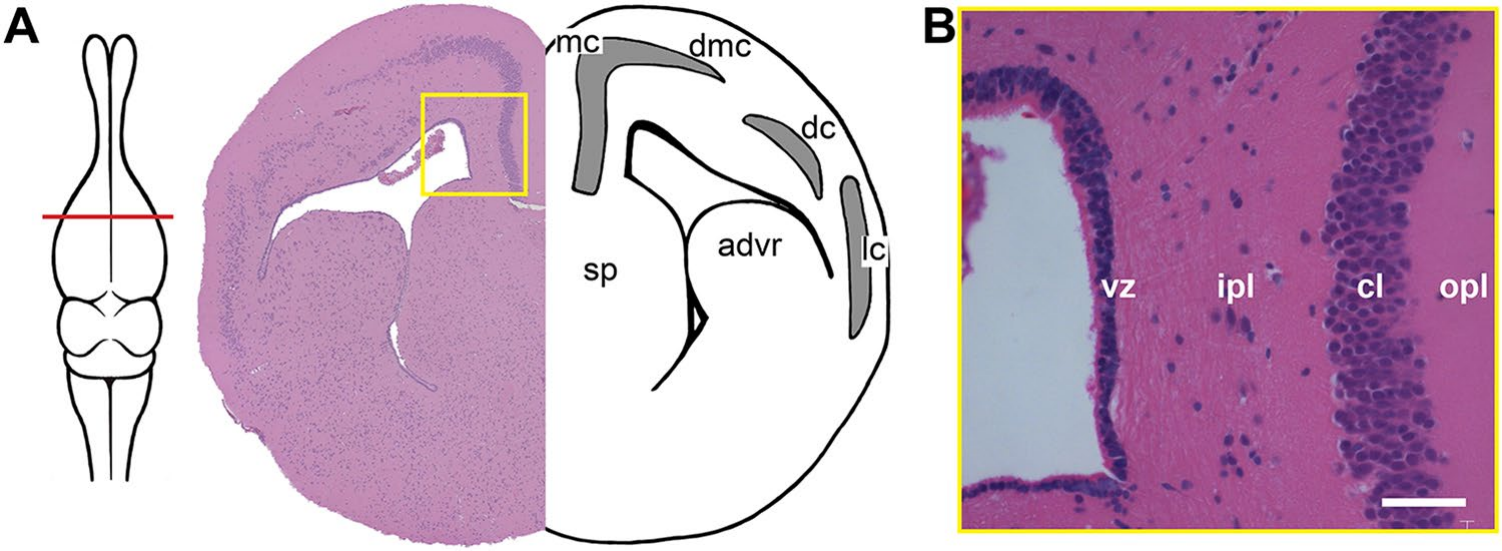
Anatomy of the sulcus septomedialis and medial cortex. (**A**) Transverse section through the telencephalon, stained with hematoxylin and eosin (red line = level of section), yellow box indicates position of (**B**). (**B**) Location of the sulcus septomedialis and medial cortex. The ventricular zone of the sulcus septomedialis is separated from the neuron-rich cellular layer of the medial cortex by a cell-sparse inner plexiform layer. The outer plexiform layer separates the cellular layer from the pial surface of the brain. Scale bar: 20 μm. advr = anterior dorsal ventricular ridge, cl = cellular layer, dc = dorsal cortex, dmc = dorsal medial cortex, ipl = inner plexiform layer, lc = lateral cortex, mc = medial cortex, opl = outer plexiform layer, sp = septum, vz = ventricular zone.

The medial cortex, situated at the dorsomedial border of the cerebral hemisphere, is thought to play an important role in place learning and relational memory^37^. As for other lizard species, the medial cortex demonstrates a three-layered organization identical to that of the dorsal and lateral cortices: a cell-dense cellular layer nested between cell-sparse inner and outer plexiform layers (Fig. 1A,B). While the outer plexiform layer borders the pial surface, the inner plexiform layer adjoins the cell population contacting the lateral ventricles known as the ventricular zone. The cellular layer demonstrates two morphologically distinct populations of neurons, small type I cells with limited cytoplasm, and larger type II cells with abundant cytoplasm^14^. On the basis of these cell types, the cellular layer is sometimes subdivided into the medial cortex (type I cells) and dorsomedial cortex (type II cells). For the purpose of this investigation, we focused solely on type I cells of the medial cortex.

### Radial glia are present within the ventricular zone

**Figure 2:**
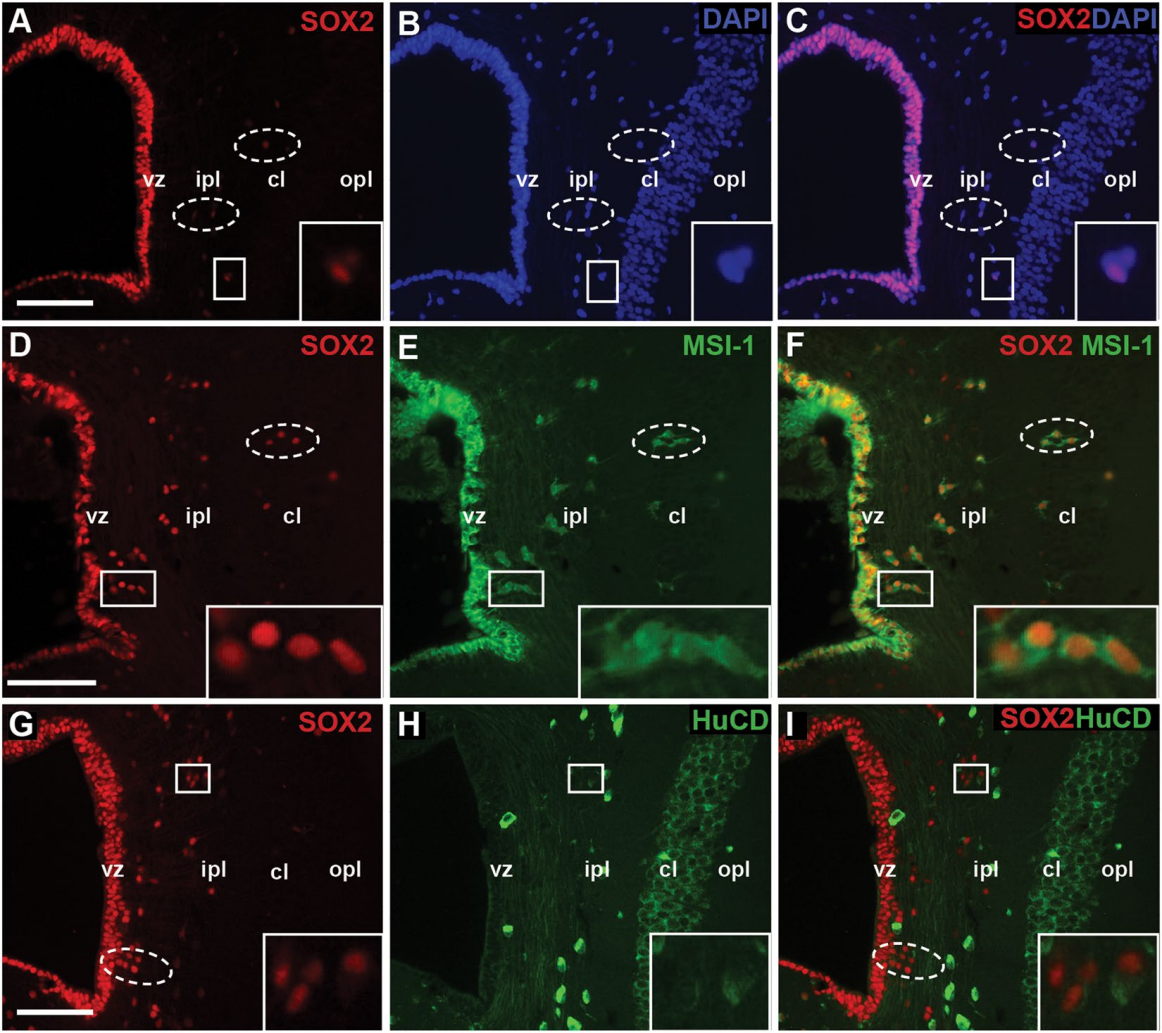
SOX2, Musashi-1 and HuC/D expression in the ventricular zone and cellular layer. (**A**–**C**) Ventricular zone cells ubiquitously express the NSPC marker SOX2. A subset of SOX2+ cells are also observed within the inner plexiform (hatched ellipses and inset), but not cellular layers. (**D**–**F**) Ventricular zone cells co-express NSPC marker Musashi-1. SOX2+/MSI-1+ cells are also present within the inner plexiform layer, often appearing in chains or with their long axes perpendicular to the ventricular lumen (hatched ellipses and inset). The shape, position, and immunoreactivity of these cells are consistent with their identification as neuroblasts. (**G**–**I**) Double immunofluorescence for SOX2 and neuronal marker HuC/D revealed that SOX2+/HuCD− (hatched ellipse), SOX2+HuCD+ (inset), and SOX2-HuCD+ cells reside within the inner plexiform layer. In contrast, the cellular layer is dominated by SOX2−/HuCD+ cells. All scale bars: 15 μm. cl = cellular layer, ipl = inner plexiform layer, opl = outer plexiform layer, vz = ventricular zone, white boxes = high magnification insets.

To identify neural stem/progenitor cells (NSPCs) within the ventricular zone, we first immunostained with SOX2. SOX2 is a hallmark transcription factor of stemness, and is involved in the induction and maintenance of pluripotency^38,39^. Across species, SOX2 identifies NSPCs within the brain during both embryogenesis and adulthood^40^. We determined that all ventricular zone cells of the telencephalon demonstrated robust SOX2 expression (Fig. 2A–C). Additional SOX2 labeling was observed among a subset of cells in the inner plexiform layer, but very rarely within the cellular layer. To expand our NSPC panel, we next co-localized SOX2 with the RNA binding protein Musashi-1 (MSI-1). MSI-1 has been implicated in asymmetric cell division and (within the brain) mitotically active stem cell populations^41,42^. Supporting their identification as an NSPC population, we observed strong SOX2/MSI-1 co-localization by all cells of the ventricular zone (Fig. 2D–F). Our data also revealed a population of SOX2+/MSI-1+ cells within the inner plexiform layer. Adjacent to the ventricular zone these double-labeled cells were organized into chains. However, as they approached the cellular layer, they were oriented with their long axes perpendicular to the ventricular surface, consistent with their identification as immature migrating neurons (i.e. neuroblasts). Next, we extended our characterization to include the RNA binding protein HuC/HuD (HuCD), a marker common to both immature and mature neurons (Fig. 2G–I). HuCD expression was varied across the medial cortex. In the ventricular zone, cells were uniformly SOX2+/HuCD−. Beginning at the interface of the ventricular zone and inner plexiform layer, we first observed HuCD+ cells. Within the inner plexiform layer three distinct cell types were observed: SOX2+/HuCD−, SOX2−/HuCD+, and (less commonly) SOX2+/HuCD+. Notably, SOX2+/HuCD+ cells of the inner plexiform layer were more fusiform and less immunoreactive for HuCD than rounded SOX2−/HuCD+ cells. In contrast, the cellular layer was dominated by SOX2-/HuCD+ cells.

**Figure 3:**
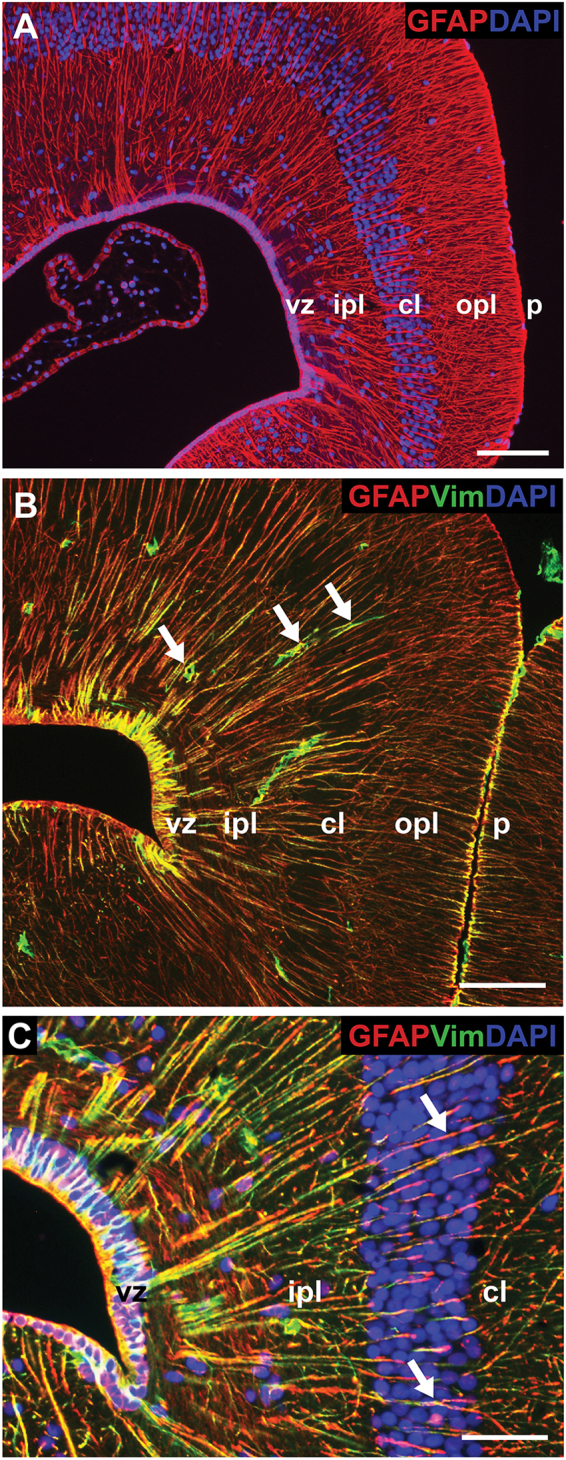
Ventricular zone cells express radial glial markers glial fibrillary acidic protein (GFAP) and Vimentin. (**A**) Cells of the ventricular zone are ubiquitously GFAP+, and extend lengthy basal processes that span the cortex to terminate at the pial surface of the brain. (**B**) Ventricular zone cells and processes additionally express developmental radial glial marker Vimentin. Vimentin staining also reveals endothelial cells of blood vessels (white arrows). (**C**) GFAP+/Vimentin+ processes can be unambiguously traced from the ventricular zone through the inner plexiform layer and cellular layer (white arrows). Scale bar A,B: 20 μm, C: 10 μm. cl = cellular layer, ipl = inner plexiform layer, opl = outer plexiform layer, p = pial surface, vz = ventricular zone.

Amongst adult reptiles, NSPCs of the ventricular zone are most commonly identified as radial glia^14,17^. To determine if radial glia were present in leopard geckos, we first used a classic marker of this cell type, the intermediate filament glial fibrillary acidic protein (GFAP). Virtually all cells of the ventricular zone were GFAP+ (Fig. 3A). Moreover, these cells demonstrated the characteristic morphology of radial glia, contacting both the ventricular lumen and, via a lengthy radial (basal) process, the pial surface or the perivascular membranes surrounding blood vessels. Radial processes passed directly through the cellular layer often bifurcating in the outer plexiform layer before contacting the pial surface. We corroborated our GFAP data using a second radial glia marker, the intermediate filament Vimentin, which is most widely recognized as a neurodevelopmental marker. Cell bodies in the ventricular zone and radial processes were strongly and ubiquitously Vimentin+/GFAP+ (Fig. 3B,C). Although Vimentin immunostaining additionally revealed the endothelium of blood vessels, clear contact between Vimentin+/GFAP+ end feet and Vimentin+ vessels could not be discerned. It is also worth noting we did not observe any stellate-shaped GFAP+ or Vimentin+ astrocytes within the cortex.

**Figure 4:**
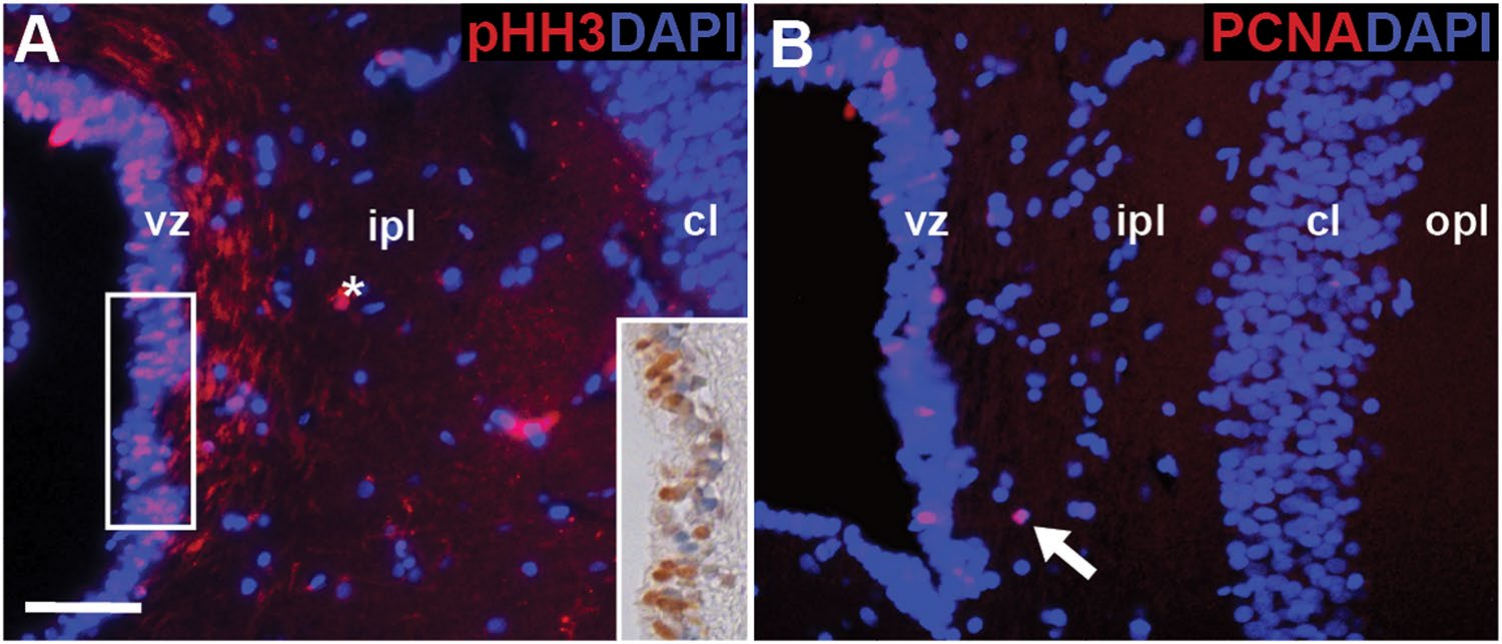
Ventricular zone cells express proliferation markers phosphorylated histone -H3 (pHH3) and proliferating cell nuclear antigen (PCNA). (**A**) Within the sulcus septomedialis, pHH3+ cells are restricted to the ventricular zone. Expression was confirmed using immunohistochemistry (inset). (**B**) Likewise, PCNA+ cells are most abundant in the ventricular zone, although occasionally observed in close contact to the ventricular zone within the inner plexiform layer (white arrow). Neither pHH3+ nor PCNA+ cells were ever observed within the cellular layer. Asterisk indicates an artifact. Scale bar A: 10 μm. cl = cellular layer, ipl = inner plexiform layer, opl = outer plexiform layer, vz = ventricular zone.

Another characteristic feature of radial glia is that they are constitutively active. To identify proliferative activity in the gecko cerebral cortex, we used the M-phase marker phosphorylated histone-H3 (pHH3), and the S-phase marker proliferating cell nuclear antigen (PCNA). pHH3+ cells were restricted exclusively to the ventricular zone (Fig. 4A). Similarly, PCNA immunostained cells were most abundant in the ventricular zone, although several positive cells were also located in the inner plexiform layer in close proximity to the ventricular zone (Fig. 4B). In stark contrast, pHH3+ or PCNA+ cells were never observed within the cellular layer of the medial cortex.

### A supportive microenvironment

**Figure 5:**
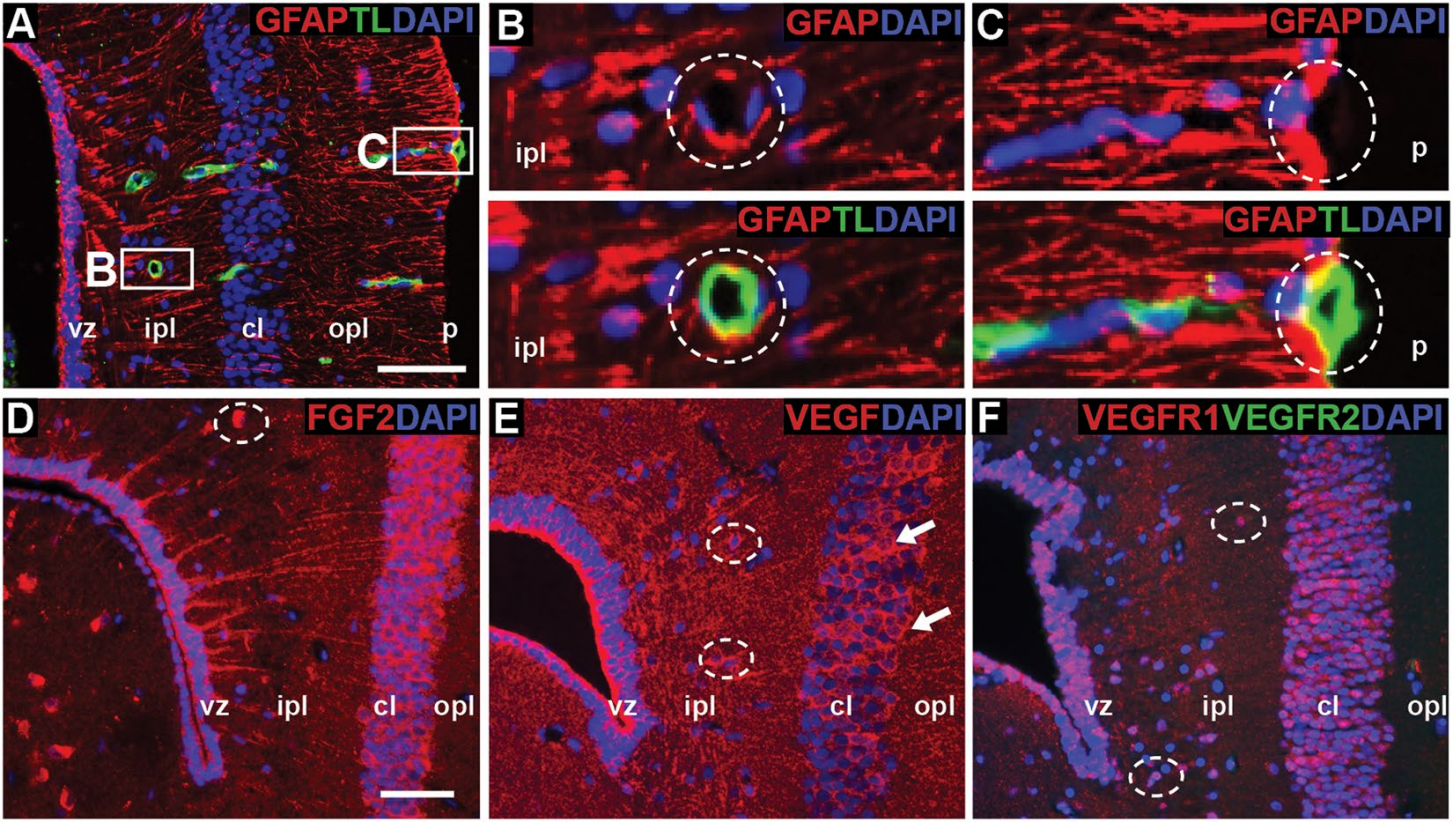
Microenvironment and associated vasculature of the ventricular zone. (**A**) Tomato lectin (TL) expressing blood vessels are cut in transverse in close proximity to the ventricular zone, and in longitudinal directed toward the pial surface. All TL+ vessels appear to be surrounded by GFAP+ radial glia endfeet (**B**,**C**). (**D**,**E**) The ventricular zone expresses both fibroblast growth factor-2 (FGF2) and vascular endothelial growth factor (VEGF). Subsets of FGF2+ and VEGF+ cells are also identified within the inner plexiform layer (hatched ellipses), and are abundant within the cellular layer (white arrows in E). (**F**) Investigating VEGF receptors, we found populations of VEGFR1+ cells within the ventricular zone, inner plexiform layer (hatched ellipses) and cellular layer. All cells were VEGFR2-. Scale bar A:15 μm, D: 10 μm. cl = cellular layer, ipl = inner plexiform layer, opl = outer plexiform layer, p = pial surface, vz = ventricular zone.

Next, we sought to characterize the microenvironment associated with the ventricular zone, with a focus on vasculature. Previous studies in mammals have revealed a relationship between blood vessels and vascular-derived factors, and neurogenesis^43–47^. We reasoned that a comparable vascular niche should also exist in neurogenic regions of the gecko brain. We co-localized GFAP, a marker of radial glia, with tomato lectin (TL), a carbohydrate binding protein common to microglia and blood vessels. Matching our previous findings with Vimentin, we observed TL+ blood vessels throughout the telencephalon (Fig. 5A). Blood vessels were conspicuously arranged so that those at the pial surface were oriented radially, whereas closer to the ventricular lumen they were cut in transverse. GFAP+ end-feet appeared to surround the lumen of all TL+ blood vessels (Fig. 5B,C).

We then explored the expression pattern of two growth factors involved in angiogenesis: basic fibroblast growth factor (FGF2) and vascular endothelial growth factor A (VEGF). Along with their pro-angiogenic roles, both FGF2 and VEGF are known to maintain NSPC pools, and to be potent mitogenic and neurogenic factors *in vivo* and *in vitro*^43,48–50^. Although the telencephalon is enriched in both FGF2 and VEGF, expression is most pronounced within the ventricular zone of the sulcus septomedialis (Fig. 5D,E). Both growth factors were also expressed by a subset of cells within the inner plexiform layer, and most cells within the adjacent cellular layer. In the mammalian dentate gyrus of the hippocampus, VEGF signalling by NSPCs occurs primarily via VEGFR2^48,51^. However, in leopard geckos, co-localization of VEGFR1 and VEGFR2 show the ventricular zone to house a population of VEGR1+ cells but to be uniformly VEGFR2- (Fig. 5F). A subset of cells within the inner plexiform layer, and the majority of cells within the cellular layer are also VEGFR1+/VEGFR2−.

### Cells generated in the ventricular zone become neurons in the cellular layer

**Figure 6:**
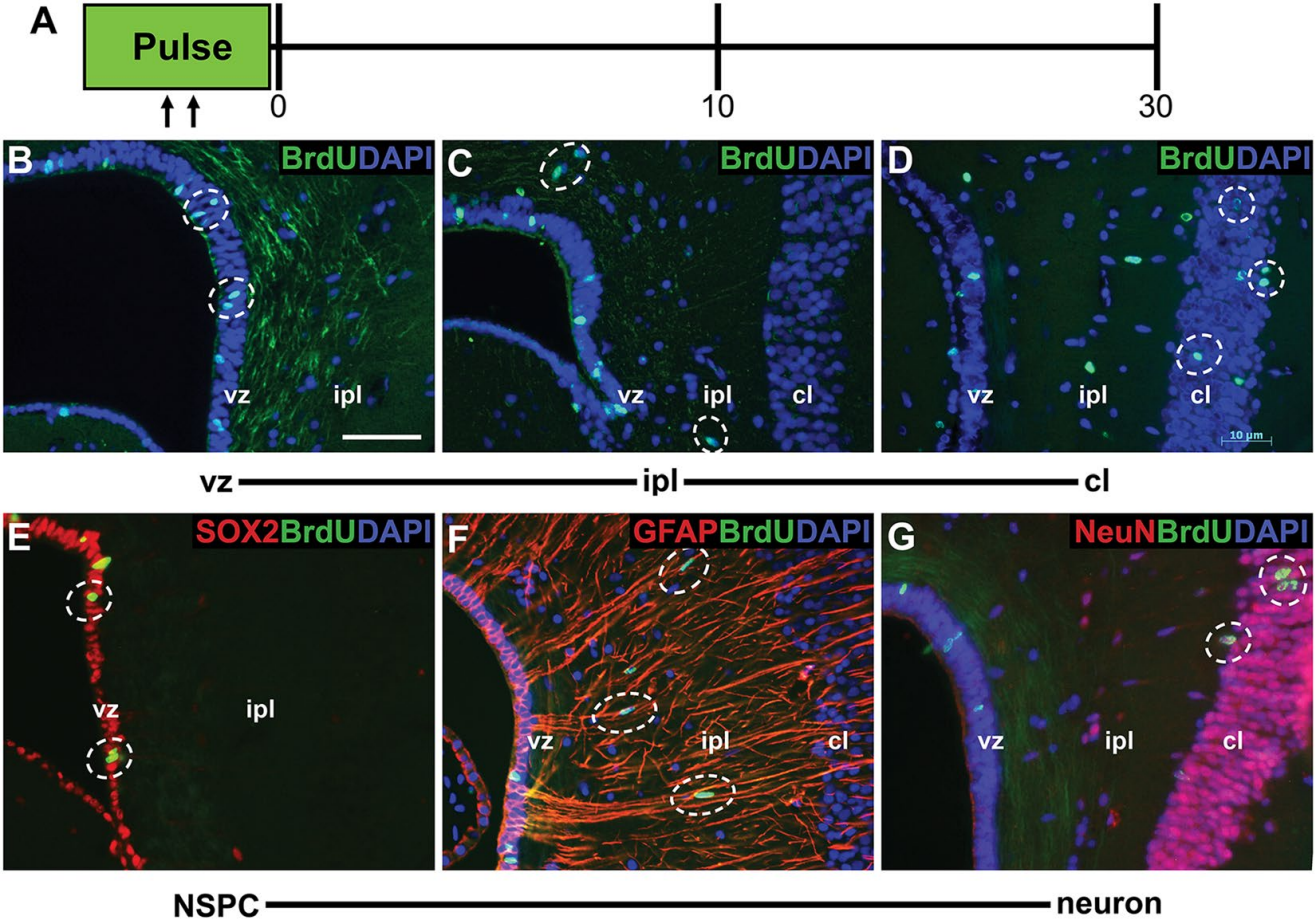
Cells generated by the ventricular zone become neurons in the cellular layer. (**A**) Experimental design for the 5-bromo-2′-deoxyuridine (BrdU) cell tracking experiment. BrdU was injected intraperitoneally twice daily for two days (pulse). Geckos were collected at days 0, 10, and 30 following the pulse. BrdU+ cells (hatched ellipses) were restricted to the ventricular zone at day 0 (**B**), had migrated into the inner plexiform layer by day 10 (**C**), and were observed in the cellular layer by day 30 (**D**). At day 0 (**E**), BrdU+ cells were of the ventricular zone co-localized with SOX2. By day 10 **(F**), BrdU+ cells within the inner plexiform layer were closely associated with GFAP+ radial processes. At day 30 (**G**), BrdU+ cells in the cellular layer co-localized with mature neuronal marker NeuN. Scale bar: 15 μm. cl = cellular layer, ipl = inner plexiform layer, vz = ventricular zone.

To track the fate of newly generated cells, we conducted an acute 5-bromo-2′-deoxyuridine (BrdU) pulse-chase experiment. BrdU is a thymidine analogue that is incorporated into cells during DNA synthesis^52^. Subsequently, cells that have incorporated the BrdU label can be visualized using immunofluorescence. We administered BrdU (intraperitoneal injection; 50 mg/kg) twice daily for a 2-day pulse period. Geckos were collected at three chase time points: day 0 (immediately following the pulse), day 10 post-pulse, and day 30 post-pulse (Fig. 6A). At day 0, BrdU+ cells were restricted to the ventricular zone, indicating that proliferation was localized to this population during the pulse period (Fig. 6B). At day 10, BrdU+ cells were identified within both the ventricular zone and the inner plexiform layer, demonstrating that cells born in the ventricular zone had begun to migrate (Fig. 6C). At day 30, BrdU+ cells were located within each of the ventricular zone, inner plexiform layer, and their presumptive final destination, the cellular layer of the medial cortex (Fig. 6D).

To explore the identity of these BrdU+ populations we conducted a series of co-localizations using SOX2, GFAP, and the mature neuronal marker NeuN. At each chase time point (day 0, 10, and 30) all BrdU+ cells in the ventricular zone co-localized with SOX2 (Fig. 6E, Fig. S1). At day 10, BrdU+ cells within the inner plexiform layer were fusiform in shape, SOX2- and NeuN-, and demonstrated a close association with GFAP+ radial processes (Fig. 6F). Specifically, the long axis of BrdU+ cells was arranged exactly parallel to that of the GFAP+ processes. By day 30, all BrdU+ cells present within the cellular layer of the medial cortex co-expressed NeuN (but neither GFAP nor SOX2) (Fig. 6G).

**Figure 7:**
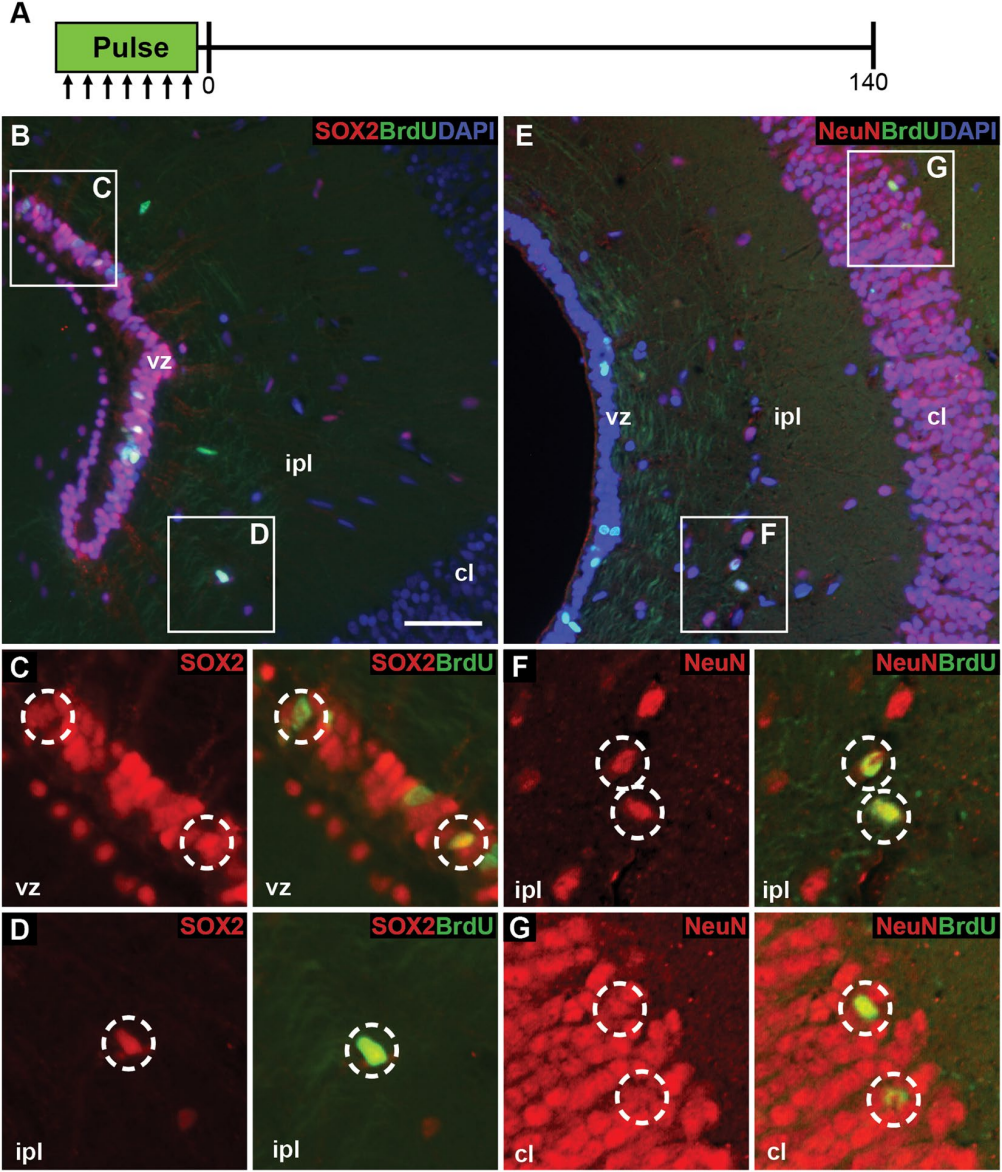
Newly generated neuronal cells persist long-term. (**A**) Experimental design for the long-duration 5-bromo-2′-deoxyuridine (BrdU) cell tracking experiment. BrdU was injected intraperitoneally twice daily for seven days (the pulse). Experimental geckos were collected immediately following, and at 140 days post-pulse. (**B**) At day 0, BrdU+ cells were located in the ventricular zone and the inner plexiform layer. All BrdU+ cells of the ventricular zone co-localized with SOX2 (**C**). A subset of BrdU+ cells in the inner plexiform layer also co-expresses SOX2 (**D**). (**E**) At day 140, BrdU+ cells were located in the ventricular zone, inner plexiform layer and cellular layer. All BrdU+ cells in the inner plexiform (**F**) and cellular layers (**G**) were NeuN+. BrdU+ cells in the ventricular zone were NeuN-. Scale bar: 15 μm. cl = cellular layer, ipl = inner plexiform layer, vz = ventricular zone.

Finally, we sought to determine if there was evidence for long-term survival of newly generated cells. Continuous cell proliferation within the ventricular zone raises the question of whether the newly generated cells are retained, and possibly incorporated into the existing neurocircuitry, or if these cells routinely undergo apoptosis and are eliminated. To identify long-term surviving cells, we used a long duration BrdU pulse-chase experiment. Geckos were administered BrdU (intraperitoneal injection twice daily; 50 mg/kg) for a 7-day pulse period, and collected at day 0 (immediately following the pulse) and day 140 post-pulse (Fig. 7A). As previously demonstrated, at day 0 BrdU+ cells were abundant within the ventricular zone, and all co-expressed SOX2 (Fig. 7B,C). In addition, likely due to the extended pulse period, a subset of BrdU+ cells were identified within the inner plexiform layer suggesting that they had begun migration. Within the inner plexiform layer, some BrdU+ cells were additionally SOX2+ (typically those in closer proximity to the ventricular zone) (Fig. 7D). By day 140, BrdU+ cells were identified within each of the ventricular zone, inner plexiform layer and cellular layer of the medial cortex (Fig. 7E). Moreover, all BrdU+ cells in both the inner plexiform layer and cellular layer co-expressed NeuN (Fig. 7F,G).

### Cell proliferation in the medial cortex is not altered in response to tail loss

**Figure 8:**
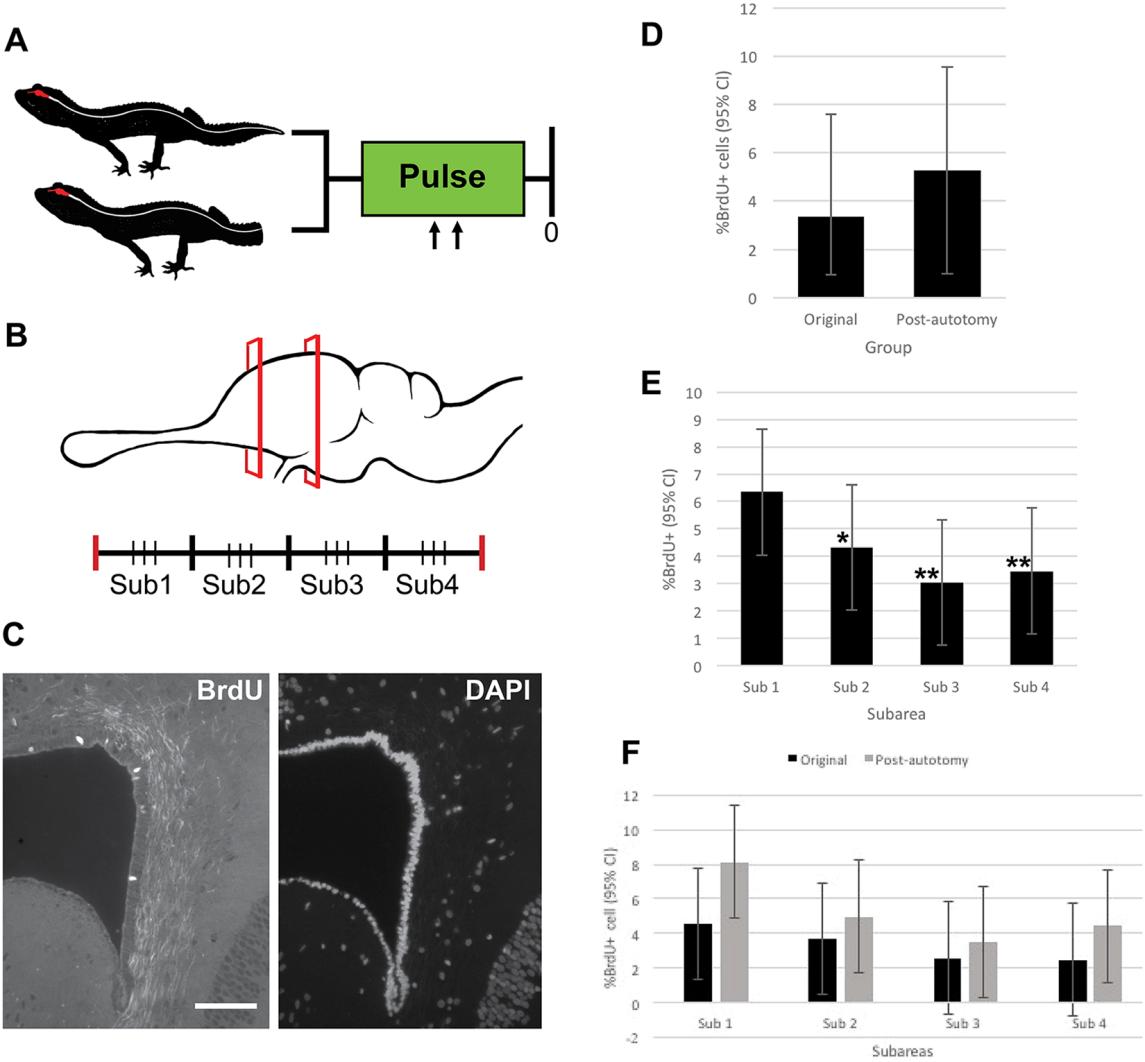
5-bromo-2′-deoxyuridine (BrdU) incorporation is not altered by tail autotomy. (**A**) Experimental design. Geckos with original (intact) or autotomized tails were injected with BrdU (pulse). All geckos were collected immediately following the pulse. (**B**) Brains were serially sectioned through the cerebral hemispheres and divided into four subareas. From each subarea, three sections were selected and immunostained for BrdU. (**C**) A BrdU to DAPI ratio was established for each section. (**D**) No significant difference in BrdU incorporation was observed between original and post-autotomy geckos (p = 0.42). (**E**) Considering original-tailed and autotomized geckos together, a rostrocaudal difference in BrdU uptake was observed. Subarea 1 took up significantly more BrdU than all other subareas (*p = 0.0002, **p < 0.0001). (**F**) For each subarea, no significant differences were observed between the two groups. Scale bar: 15 μm. CI = confidence interval, sub = subarea.

A hallmark of postnatal neurogenesis is its sensitivity to physiological and pathological stimuli, including central nervous system injuries^53,54^. Curiously, leopard geckos (and many other lizard species) spontaneously rupture their tail spinal cord during tail loss (caudal autotomy), a naturally evolved predation avoidance behaviour^29–33^. We took advantage of this reflexive ability to determine if tail spinal cord injury had an acute influence on cell proliferation in the medial cortex. We compared BrdU uptake following a 2-day pulse between post-autotomy geckos (n = 3) and original-tailed controls (n = 3) (Fig. 8A). All geckos were sacrificed 12 hours after the last BrdU administration. Focusing on the ventricular zone of the sulcus septomedialis, we quantified the number of BrdU+ cells and the total number of DAPI+ cells for both groups of geckos. For these analyses the medial cortex was serially sectioned in the transverse plane, and the histological series was divided into four equal subareas allowing for observation of any rostrocaudal differences in BrdU uptake (Fig. 8B). The data set included three sections (between 30 μm and 60 μm apart) per subarea, for a total of 12 sections per gecko. The ventricular zone of the sulcus septomedialis was imaged bilaterally (one field of view per hemisphere).

Consistent with our previous findings, in all sections examined for both experimental groups, BrdU+ cells were restricted to the ventricular zone (Fig. 8C). In the original-tailed group, 3.33% were BrdU+ (with a 95% confidence interval (CI) [lower limit = 0.95%, upper limit = 7.60%]), whereas in the post-autotomy group, 5.26% of cells were BrdU+ (with a 95% CI [lower limit = 0.99%, upper limit = 9.54%]) (Fig. 8D). Comparing the groups, we observed no significant difference in BrdU incorporation (p = 0.42), indicating that cell proliferation within the medial cortex is resilient to the effects of spinal cord rupture following tail loss.

Curiously, there did appear to be rostrocaudal differences in BrdU incorporation. Considering both groups simultaneously, BrdU uptake was significantly higher in the rostral-most subarea (subarea 1) than in all other subareas (compared to: subarea 2, p = 0.002; subarea 3 and 4, p < 0.001) (Fig. 8E). To examine if there was any effect of tail-loss specific to different subareas we conducted post-hoc Tukey pairwise comparisons. As the most constitutively active region of the sulcus septomedialis, it stands to reason that the rostal-most subarea may be the most affected following spinal cord rupture. We determined that 8.13% of cells were BrdU+ in the post-autotomy group (with a 95% CI [lower limit = 4.89%, upper limit = 11.37%]), and 4.58% of cells were BrdU+ in the original-tailed group (with a 95% CI [lower limit = 1.34%, upper limit = 7.81%]) (Fig. 8F). Consistent with the results achieved by surveying the entirety of the medial cortex, differences in BrdU incorporation were not statistically significant (p = 0.12).

## Discussion

This study provides the first evidence of postnatal neurogenesis in a representative, commercially bred lizard species, the leopard gecko. Using a BrdU pulse-chase strategy and a panel of protein markers, we determined that cells from the ventricular zone of the sulcus septomedialis constitutively proliferate and contribute new neurons to the medial cortex, homologue of the mammalian hippocampal formation^18–20^. Our data also show that the sulcus septomedialis is a neurogenic niche, complete with an associated vascular compartment. Finally, we found that cell proliferation within the medial cortex is not significantly altered in response to rupture of the tail spinal cord (as a result of autotomy). Combined, our findings reveal that leopard gecko represents a laboratory-appropriate reptilian model to explore the regenerative capacity of the central nervous system.

As for other lizard species, and non-mammalian vertebrates in general, we identified radial glia within the ventricular zone. Consistent with this interpretation, we determined that cells of the ventricular zone robustly co-express the NSPC markers SOX2 and MSI-1, as well as the intermediate filaments GFAP and Vimentin. GFAP/Vimentin co-localization was particularly prominent within the lengthy radial processes. These data are further supported by a previous investigation using leopard geckos that also observed radial processes within the brain to be immunoreactive for GFAP and Vimentin (although they did not test for co-expression^55^), as well as the recent documentation of this cell type (with a similar protein expression profile) within the spinal cord of the tail^30^. Vimentin is typically associated with developmental immaturity in mammals and other  vertebrates^56,57^. Hence, it is possible that Vimentin expression by radial glia in leopard geckos is an example of paedomorphosis: a juvenile trait retained into adulthood. However, until more widespread taxonomic sampling has been conducted, the significance of this feature remains unclear.

Another key feature of radial glia is homeostatic proliferation. Using the S-phase marker PCNA, late G2/M-phase marker pHH3, and an acute (2-day) pulse with BrdU, we determined that the sulcus septomedialis is the sole anatomical locus of proliferation in the vicinity of the medial cortex. Although occasional PCNA+ cells were observed within the inner plexiform layer, they were always located near the ventricular zone. Furthermore, similarly positioned cells were always immunonegative for pHH3 and BrdU incorporation. By way of explanation, we note that PCNA is present for a longer timeframe following cell division than pHH3^58,59^. These findings also confirm that a subventricular zone, comparable to that seen in mammals, is absent from the postnatal lizard brain. Although not quantified, the relative abundance of pHH3+ cells appears to be greater than that of PCNA+ cells, which may offer insight into cell-cycle dynamics of the ventricular zone. The unexpected abundance of pHH3+ as compared to PCNA may be a unique feature of the lizard model. Previous work has shown that cortical progenitors in embryonic lizards spend a smaller proportion of their cell cycle in S-phase than their mammalian counterparts^60^. Alternatively, it may be a conserved feature of NSPCs across taxa. Mammalian NSPCs have also been reported to show lengthening of M-phase/shortening of S-phase (as compared to other cell types)^61,62^. Specifically, in adult mice radial glia-like B1 cells have a surprisingly short S-phase (only ~4 hours), in comparison to G2/M phase (~6 hours)^62^. This altered cell-cycle has been proposed as a mechanism for control and regulation of proliferation, and a determinant of cell fate.

Although the taxonomic sample size remains small, it is worth noting that our experiments with the leopard gecko correspond well with data reported for other lizard species. For example, as for most lizards – including the gecko *T*. *mauritanica*^16^, the gallotine lacertids *G*. *galloti* and the Algerian Psammodromus, *Psammodromus algirus*^26,63^, and the tropodurid iguanian *T*. *hispidus*^15^ – the timeframe for neuroblast migration into the medial cortex of the leopard gecko is ~30 days. While electrophysiological confirmation remains to be performed, we (and others^15,16^) have determined that upon arriving, these labeled cells have adopted a neuronal phenotype. Notwithstanding these similarities, there are several obvious examples of species-specific variation. In the lacertine lacertid *P*. *hispanicus*, it only takes seven days for cells labeled within the sulcus septomedialis to migrate to the medial cortex^27^. And whereas the duration of neuroblast migration in *G*. *galloti* matches that of leopard geckos (and most other species), it takes another two months for the newly arrived cells to differentiate into neurons (on the basis of ultrastructure^26^). The absence of a clear phylogenetic pattern to explain the neurogenic diversity of lizards underscores the importance of future comparative investigations.

Taken together, our data add new details to the existing model of lizard neurogenesis, with important implications for all vertebrates. The ventricular zone, particularly the ependymal sulci, demonstrates all the classic features of a neurogenic niche populated by radial glia. As evidenced by a distinct panel of markers (including SOX2, MSI-1, GFAP, and Vimentin), radial glia are present, and pools of these cells are constitutively active. The ventricular zone generates SOX2+/MSI-1+ neuroblasts, which then migrate through the inner plexiform layer along GFAP+/Vimentin+ radial processes. During this migration, neuroblasts gradually transition from a strongly neurogenic phenotype (SOX2+/MSI-1+/HuCD−/NeuN−) at the ventricular zone, to one that is characteristically neuronal (SOX2-/MSI-1-/HuCD+/NeuN+) as they approach the medial cortex. In some respects, this neurogenic to neuronal transformation closely parallels that observed in the rostral migratory stream of mice, as neuroblasts migrate from the subventricular zone of the lateral ventricles to eventually populate, as mature neurons, the olfactory bulbs^64^. Ultimately, newly generated cells originating in the ventricular zone come to reside either in the inner plexiform layer (as interneurons) or the cellular layer of the medial cortex, where they persist long-term as NeuN+ and HuCD+ neurons.

The abundant neurogenic capacity and long-term survival of neurons generated from the sulcus septomedialis are likely due, at least in part, to the supportive microenvironment of this anatomical region. Matching the neurogenic microenvironments observed in mammals, we determined that direct contact with vasculature (via radial glia end feet) and pro-angiogenic growth factors are characteristic of neuron-forming and neuron-supporting compartments in leopard geckos^45,47,48,65^. VEGF and FGF2 are well-described in neurogenic regions of the mammalian brain^48,66–68^, but to our knowledge this is the first report of their expression in the lizard brain. Both the sulcus septomedialis and the medial cortex demonstrate robust expression of VEGF and FGF2. Although their exact role remains unclear, in mammals it has been suggested that these cytokines function in promoting neurogenesis, either directly as a mitogen or indirectly promoting the neurogenic niche, and as a neuroprotective factor within the neuron-dense cellular layer^43,48–50^. Our investigation also revealed that, unlike the dentate gyrus of mammals, the ventricular zone of leopard geckos is VEGFR1+ but VEGFR2-. This is suggestive that the neurogenic compartment of the lizard brain uses a distinct form of signalling.

Finally, we showed that cell proliferation within the medial cortex, normally associated with neurogenesis, was not impacted by tail spinal cord rupture (as a result of tail loss). More specifically, our findings revealed that there is no significant change in proliferation, evidenced by BrdU uptake, between leopard geckos within intact (original) tails and those induced to self-detach their tails. In contrast, among mammals the hippocampal formation is particularly sensitive to spinal cord injuries^36,69^. For example, in rats a partial hemisection of the cervical spinal cord results in a significant reduction in the number of BrdU+ cells within 7-days following injury^36^. We speculate that the comparative resilience of the leopard gecko medial cortex to spinal cord injuries is an adaptation associated with tail loss (caudal autotomy), functioning to minimize physiological disruption after a traumatic encounter with a predator. These findings underscore the continued significance of reptilian models to the study of neurogenesis.

## Materials and Methods

### Animal Care

Captive bred *Eublepharis macularius* (leopard geckos) were acquired from a commercial supplier (Global Exotic Pets, Kitchener, Ontario, Canada). At the beginning of the experiment, all animals were sexually immature and less than one year old. Growth was monitored throughout the experimental period by measuring mass and snout-vent length weekly. Animal Usage Protocols (AUPs) were approved by the University of Guelph Animal Care Committee (protocol #1954) and are in accordance with the procedures of the Canadian Council on Animal Care. Geckos were individually housed according to the protocols of McLean and Vickaryous^31^, in an isolated, temperature-controlled environmental chamber (average ambient temperature 27.5 °C; photoperiod 12:12). Geckos were fed 3 larval *Tenebrio* spp. (mealworms) dusted with powdered calcium and vitamin D3 (cholecalciferol) (Zoo Med Laboratories Inc., San Luis Obispo, California, USA) daily, and had free access to clean drinking water.

### Tail Autotomy

Tail autotomy, the voluntarily detachment of a portion of the tail, is a naturally evolved an anti-predation strategy common to many lizard species. As reported elsewhere^29–31^, autotomy is well tolerated by leopard geckos. To initiate autotomy, geckos were manually restrained (without the use of anesthesia) and, using the thumb and index finger, firm and continuous pressure was applied at the first tail segment distal to the pelvis. Tails then detached at the level of applied pressure. All geckos survived the autotomy procedure, and resumed normal behaviours, including feeding, within one day.

### 5-bromo-2′-deoxyuridine (BrdU) Injections

All BrdU experiments were conducted using the protocol of Gilbert and Vickaryous^30^. BrdU is a thymidine analog that is incorporated into DNA during the synthesis (S) phase of the cell cycle^70,71^. During BrdU administration (the pulse) cycling cells incorporate the analogue, which is then detected using immunofluorescence at subsequent time-points (the chase). BrdU+ cells continue to pass the analogue to daughter cells until such time that the signal falls below the threshold of immunodetection.

A 50 mg/mL BrdU stock solution was prepared by diluting 50 mg of BrdU powder (Sigma- Aldrich, St. Louis, Missouri, USA) in 1 mL dimethyl sulphoxide (DMSO). A working solution (5 mg/mL) was then prepared by diluting 1 mL of the stock in 9 mL of sterile 1x phosphate buffered saline (PBS). The working BrdU solution was injected at a dose of 50 mg/kg twice daily at 12-hour intervals (9 am and 9 pm) using a 0.5cc insulin syringe (Abbott Laboratories, Saint-Laurent, Quebec, Canada) into the peritoneal cavity. This dosage is commonly used for studies of adult neurogenesis^72,73^, and has been shown to be non-toxic^74^. Left and right-sided injections were alternated at each injection interval.

A short duration BrdU pulse-chase experiment was performed to document proliferation, and to track the migration and fate of newly generated cells. Experimental geckos were administered BrdU during a two-day pulse (four injections at 12 hour intervals), and collected at three chase time points (Tables S1-S4): chase day 0, 12 hours following the last BrdU injection (n = 3); chase day 10 (n = 3); and chase day 30 (n = 3).

To determine if neurogenesis is altered in response to tail autotomy, we conducted a parallel short duration BrdU pulse-chase experiment, with one group of geckos induced to autotomize their tail immediately prior the first BrdU injection. As above, post-autotomy geckos were administered BrdU (4 injections at 12 hour intervals), and collected at chase day 0. To reduce group differences due to relatively low sample size, all experimental geckos were first randomly assigned to groups, and then groups were balanced using weight of individual geckos (as a proxy for size and age).

To quantify the proportion of cycling cells, brains were collected at chase day 0 from original tailed (n = 3) and post-autotomy (n = 3) geckos, and serially sectioned (transverse plane) through the medial cortex. We landmarked our region of interest (ROI; the sulcus septomedialis) using the first appearances of the anterior dorsal ventricular ridge (representing the rostral margin) and the third ventricle (the caudal margin). Each ROI was then longitudinally divided into four subareas, each containing an equal number of sections, to investigate any potential differences in cell proliferation across the rostrocaudal axis. From each subarea, three sections (between 30 to 60 μm apart) were selected (=12 sections/gecko). The sulcus septomedialis from the right and left sides were imaged using the 40x objective of an Axio Imager D1 Microscope (Carl Zeiss Canada Ltd; Toronto, Canada) with an Axiocam MRc 5 camera (Carl Zeiss Canada Ltd; Toronto, Canada). ImageJ software was used to perform the image analysis. The total number of nuclei in each sulcus septomedialis for each image (as indicated by the nuclear counterstain DAPI) was counted automatically using the ImageJ-ITCN plugin (using parameters of cell length = 17 pixels, minimum distance = 8.5 pixels), while the number of proliferating cells (indicated by BrdU) was counted manually by a blinded counter (Table S5).

All analyses were performed using SAS 9.2 software (SAS Institute, Cary, North Carolina, USA). A general linear model that included the main effects of group (original tailed or post-autotomy), subarea (rostral-caudal subareas 1–4), and side of cerebral hemisphere, as well as their interactions, was used to determine if the BrdU ratio (BrdU+ cells to DAPI+ cells) was significantly different between levels of these factors. No significant difference in the BrdU ratio was found due to side of cerebral hemisphere (left or right) (p = 0.91); hence consideration of side was removed in the final model. Data was checked for normality with a Shapiro-Wilk test and examination of the residuals, and was found to be fundamentally normal. If the overall F-test was significant, post hoc Tukey pairwise comparisons are reported.

A long duration BrdU pulse-chase experiment was performed to investigate label-retaining cells (=slow-cycling), and for documentation of long-term survival of daughter cells. Experimental geckos were administered BrdU during a seven-day pulse (14 injections at 12 hour intervals), and collected at two chase time points (Tables S6,S7). The first group of geckos (n = 3) was collected 12 hours following the last BrdU injection (chase day 0). A second group of geckos (n = 3) was collected at chase day 140. One leopard gecko died of unknown causes during the experiment and was replaced.

### Tissue Collection, Preparation and Histochemistry

Geckos were euthanized by injection of 150 μL alfaxalone into the hypaxial muscles of the neck and, following loss of righting reflex, exsanguinated (transcardial perfusion with phosphate buffered saline (PBS)), followed by 10% neutral buffered formalin (NBF; Fisher Scientific, Waltham, Massachusetts, USA) and decapitation. Brains were dissected out and post-fixed by submersion in 10% NBF for an additional 22 hours. Tissues were transferred to 70% ethanol prior to processing. Using an automated processor (Fisher Scientific, Waltham, Massachusetts, USA), tissues were dehydrated, clearing in xylene, and infiltrated with paraffin wax. Tissue samples were then embedded transversely in paraffin blocks and sectioned at 5 μm using a rotary microtome (Shandon Finesse ME+, Thermo Fisher Scientific), mounted on charged slides (Surgipath X-tra, Leica Microsystems, Concord, Ontario, Canada), and baked at 60 °C overnight. To investigate brain structure and tissue architecture, representative sections were stained with Hematoxylin and Eosin (File S1).

### Immunofluorescence

For antibody details, please see Table 1. A Standardized Protocol was performed to visualize SOX2, Musashi-1, GFAP, Vimentin, HuC/D, tomato lectin, BrdU and NeuN (File S1). Briefly, sections were de-paraffinized, rehydrated to water, rinsed in PBS, and subject to one of two antigen retrieval methods: sections stained with SOX2/Musashi-1 or SOX2/HuCD were incubated for 30 minutes in 2 N hydrochloric acid at 37 °C; all other sections were submerged in citrate buffer at 95 °C for 12 minutes, and cooled for 20 minutes at room temperature (RT). Following antigen retrieval all sections were incubated for 20 minutes in 0.1% trypsin at 37 °C (Sigma-Aldrich, St. Louis, Missouri, USA), and blocked for 30 minutes at 37 °C in 5% normal goat serum (NGS) in diluent (1% bovine serum albumin, 0.5% Tween 20, 0.1% sodium azide in PBS), with rinsing steps (PBS) in between. Sections were incubated overnight at 4 °C in primary antibody diluted in diluent (SOX2 [1:50] Cell-Signalling, Whitby, Ontario, Canada; GFAP [1:400] DAKO, Glostrup, Denmark; Vimentin [1:50] Developmental Studies Hybridoma Bank, Iowa City, Iowa, USA; HuC/D [1:10] Molecular Probes, Rockford, Illinois, USA; BrdU [1:200] Developmental Studies Hybridoma Bank, Iowa City, Iowa, USA; tomato lectin [1:50] Sigma-Aldrich, St. Louis, Missouri, USA; NeuN [1:125] Abcam, Cambridge, Massachusetts, USA). One section per slide served as an omission control. The next day, sections were incubated in secondary antibody at RT (for 1 hour), stained with nuclear marker DAPI ([1:5000] Life Technologies, Eugine, Oregon, USA) (2 minutes), and then coverslipped with fluorescent mounting media (DAKO, Glostrup, Denmark), with rinsing steps (with PBS) in between.

**Table 1:** Summary table of optimized immunofluorescence protocols for proteins of interest.

Antigen	Protocol Type	Retrieval	Block	Primary	Secondary
**SOX2** (anti-Sex-determining box region Y-Box 2)	Standard	Citrate buffer for 12 min at 95 °C, 20 min at RT in solution *or* 2 N HCl, 30 min at 37 °C 0.1% trypsin in PBS for 20 min at 37 °C	5% NGS in diluent for 30 min at 37 °C	**1:50** in diluent overnight at 4 °C (Cell Signaling, 2748 S, RRID AB_823640)	**1:200** in 1XPBS for 1 hour at RT (Cy3-conjugated Goat anti-Rabbit IgG, 111-165-144, RRID AB_2338006)
**MSI-1** (anti-Musasahi-1)	Standard	2 N HCl, 30 min at 37 °C 0.1% trypsin in PBS for 20 min at 37 °C	5% NGS in diluent for 30 min at 37 °C	**1:100** in diluent overnight at 4 °C (Millipore, Cat, MABE268, RRID AB_2576205)	**1:100** in sterile 1XPBS for 1 hour at RT (Goat anti-Mouse AlexaFluor 488, Life Technologies, A11001, RRID AB_2534069)
**HuCD** (anti-human neuronal protein HuC/HuD)	Standard	2 N HCl, 30 min at 37 °C 0.1% trypsin in PBS for 20 min at 37 °C	5% NGS in diluent for 30 min at 37 °C	**1:10** in diluent overnight at 4 °C (Molecular Probes, Cat#16A11 RRID AB_221448)	**1:500** in sterile 1XPBS for 1 hour at RT (Goat anti-Mouse AlexaFluor 488, Life Technologies, A11001, RRID AB_2534069)
**GFAP** (anti-Glial Fibrillary Acidic Protein)	Standard	Citrate buffer for 12 min at 95 °C, 20 min at RT in solution 0.1% trypsin in PBS for 20 min at 37 °C	5% NGS in diluent for 30 min at 37 °C	**1:400** in diluent overnight at 4 °C (Dako, Z0334, RRID AB_10013382)	**1:1000** in 1XPBS for 1 hour at RT (Cy3-conjugated Goat anti-Rabbit IgG, 111-165-144, RRID AB_2338006)
**Vimentin** (anti-Vimentin)	Standard	Citrate buffer for 12 min at 95 °C, 20 min at RT in solution 2) 0.1% trypsin in PBS for 20 min at 37 °C	5% NGS in diluent for 30 min at 37 °C	**1:50** in diluent overnight at 4 °C (DSHB, Cat# H5, RRID AB_528506)	**1:200** in sterile 1XPBS for 1 hour at RT (Goat anti-Mouse AlexaFluor 488, Life Technologies, A11001, RRID AB_2534069)
**pHH3** (anti-phosphorylated Histone H3 (serine 10))	Abbreviated	Citrate buffer for 12 min at 95 °C, 20 min at RT in solution	5% NGS in 1XPBS for 1 hour at RT	**1:100** in1XPBS overnight at 4 °C (Cell Signaling, 3377 S, RRID AB_1549592)	**1:250** in 1XPBS for 1 hour at RT (Cy3-conjugated Goat anti-Rabbit IgG, 111-165-144, RRID AB_2338006)
**pHH3** (anti-phosphorylated Histone H3 (serine 10))	Immunohistochemistry	Citrate buffer for 12 min at 95 °C, 20 min at RT in solution	3% NGS in 1XPBS for 1 hour at RT	**1:500** in1XPBS overnight at 4 °C (Cell Signaling, 3377 S, RRID AB_1549592)	**1: 500** in 1XPBS for 1 hour at RT (Biotinylated goat anti-Rabbit, Jackson ImmunoResearch 111-066-003, RRID AB_2337966)
**PCNA** (anti-Proliferating Cell Nuclear Antigen)	Abbreviated	Citrate buffer for 12 min at 95 °C, 20 min at RT in solution	5% NGS in 1XPBS for 1 hour at RT	**1:100** in1XPBS overnight at 4 °C (Santa Cruz BioTechnology, Inc., sc-7907)	**1:200** in 1XPBS for 1 hour at RT (Cy3-conjugated Goat anti-Rabbit IgG, 111-165-144, RRID AB_2338006)
**Tomato** **Lectin**	Standard	1) Citrate buffer for 12 min at 95 °C, 20 min at RT in solution 2) 0.1% trypsin in PBS for 20 min at 37 °C	5% NGS in diluent for 30 min at 37 °C	**1:50** in diluent overnight at 4 °C (Sigma-Aldrich, Cat# L0651)	**1:500** in sterile 1XPBS for 1 hour at RT (streptavidin, AlexaFluor 488 conjugate, Life Technologies, S32354, RRID AB_2315383)
**VEGF** (anti-Vascular Endothelial Growth Factor A)	Abbreviated	Citrate buffer for 12 min at 95 °C, 20 min at RT in solution	5% NGS in 1XPBS for 1 hour at RT	**1:50** in1XPBS overnight at 4 °C (Santa Cruz BioTechnology, Inc., sc-152, RRID AB_2212984)	**1:200** in 1XPBS for 1 hour at RT (Cy3-conjugated Goat anti-Rabbit IgG, 111-165-144, RRID AB_2338006)
VEGFR1 (anti-VEGF Receptor 1/Flt-1)	Abbreviated	Citrate buffer for 12 min at 95 °C, 20 min at RT in solution	5% NGS in 1XPBS for 1 hour at RT	**1:100** in1XPBS overnight at 4 °C (Santa Cruz BioTechnology, Inc., sc-316, RRID AB_2107031)	**1:200** in 1XPBS for 1 hour at RT (Cy3-conjugated Goat anti-Rabbit IgG, 111-165-144, RRID AB_2338006)
**VEGFR2** (anti-VEGF Receptor 2/Flk-1)	Abbreviated	Citrate buffer for 12 min at 95 °C, 20 min at RT in solution	5% NGS in 1XPBS for 1 hour at RT	**1:50** in1XPBS overnight at 4 °C (Santa Cruz BioTechnology, Inc., sc-6251, RRID AB_628431)	**1:200** in sterile 1XPBS for 1 hour at RT (Goat anti-Mouse AlexaFluor 488, Life Technologies, A11001, RRID AB_2534069)
**FGF2** (anti-basic Fibroblast Growth Factor)	Abbreviated	Citrate buffer for 12 min at 95 °C, 20 min at RT in solution	5% NGS in 1XPBS for 1 hour at RT	**1:100** in1XPBS overnight at 4 °C (Santa Cruz BioTechnology, Inc., sc-79, RRID AB_631497)	**1:200** in 1XPBS for 1 hour at RT (Cy3-conjugated Goat anti-Rabbit IgG, 111-165-144, RRID AB_2338006)
**BrdU** (anti-5-Bromo-2′-deoxyuridine)	Standard	Citrate buffer for 12 min at 95 °C, 20 min at RT in solution 2) 0.1% trypsin in PBS for 20 min at 37 °C	5% NGS in diluent for 30 min at 37 °C	**1:100** in diluent overnight at 4 °C (DSHB, G3G4, RRID AB_2618097)	**1:200** in sterile 1XPBS for 1 hour at RT (Goat anti-Mouse AlexaFluor 488, Life Technologies, A11001, RRID AB_2534069)
**NeuN** (anti-Neuronal Nuclei/FOX3)	Standard	Citrate buffer for 12 min at 95 °C, 20 min at RT in solution 2) 0.1% trypsin in PBS for 20 min at 37 °C	5% NGS in 1XPBS for 1 hour at RT	**1:125** in PBST overnight at 4 °C (Abcam, ab104225, RRID_AB10711153)	**1:1000** in 1XPBS for 1 hour at RT (Cy3-conjugated Goat anti-Rabbit IgG, 111-165-144, RRID AB_2338006)

DSHB, Developmental Studies Hybridoma Bank, University of Iowa; min, minutes; NGS, normal goat serum; PBS, phosphate buffered saline; RT, room temperature.

An Abbreviated method was used to detect PCNA, pHH3, FGF2, VEGF, VEGFR1 and VEGFR2. After being brought to water, sections underwent antigen retrieval (citrate buffer at 95 °C) and were then blocked with 5% NGS at RT. Primary antibodies were diluted in PBS and applied to one section on the slide for overnight incubation at 4**°**C (pHH3 [1:100] Cell Signaling, Whitby, Ontario, Canada; PCNA [1:100], FGF2 [1:100], VEGF [1:50], VEGFR1 [1:50], VEGFR2 [1:100] Santa-Cruz Biotechnology, Dallas, Texas, USA). Sections were incubated with the secondary antibody for one hour at RT, counterstained using DAPI, and coverslipped.

## Electronic supplementary material

Supplementary data
