# Supplementary material for: Evidence for neurogenesis in the medial cortex of the leopard gecko, Eublepharis macularius
Source: Sci Rep. 2018 Jun 25;8:9648. doi: 10.1038/s41598-018-27880-6 (PMC6018638; doi:10.1038/s41598-018-27880-6)
Supplement: Supplementary file 1 — Supplementary data [file 41598_2018_27880_MOESM1_ESM.pdf]

Evidence for neurogenesis in the medial cortex of the leopard gecko, *Eublepharis macularius*

Supplementary Information

Rebecca P McDonald[1] and Matthew K Vickaryous[1*]

[1] Department of Biomedical Sciences, Ontario Veterinary College, University of Guelph, Guelph, Ontario, Canada

Corresponding author:

Matthew K. Vickaryous

E: mvickary@uoguelph.ca

SUPPLEMENTARY FIGURE

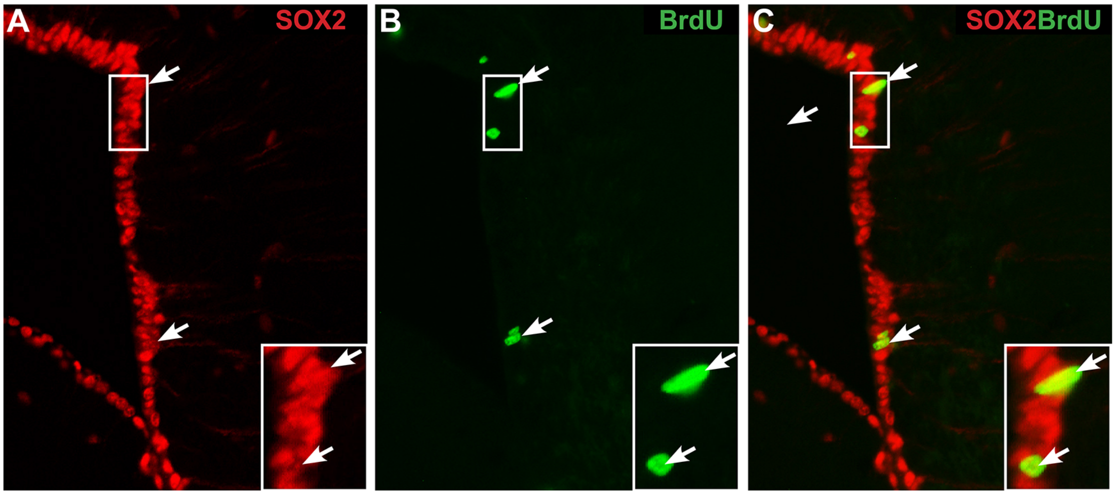

Figure S1. 5-bromo-2'-deoxyuridine (BrdU) labeled cells of the ventricular zone co-localize with SOX2. BrdU was injected intraperitoneally twice daily for two days (pulse). Geckos were collected at day 0 following the pulse. Virtually all cells of the ventricular zone express SOX2 (A). BrdU+ cells (white arrows; insets = higher magnification) were restricted to the ventricular zone at day 0 (B,C).

SUPPLEMENTARY INFORMATION

Acute and long-duration 5-bromo-2’-deoxyuridine (BrdU) pulse-chase collection time points and experimental gecko weights.

Experiment start date: Sept. 14, 2016. All geckos were subadults at the start of the experiment, with no externally visible sex characteristics. BrdU injections given i.p., 2x daily (8am, 8pm). Short pulse = 2 day pulse; long pulse = 7 day pulse. All days counted from experimental day (d) 1 (e.g., short pulse d12 = 2d pulse, 10d chase). Injection dose= mass x 10 microlitres. All tail autotomies were performed at ~8am, immediately followed by first pulse injection. All BrdU doses for autotomized geckos were calculated following tail loss.

Table S1. Leopard gecko group assignments

Gecko ID number	***Mass (g) at start***	***Snout to vent length (mm)***	***Tail length (mm)***	***Experimental group***
Ap2126	14.0	95	70	Short pulse d2, original tail
Ap2107	15.6	90	70	Short pulse d2, original tail
Ap2113	12.1	90	70	Short pulse d2, original tail
Ap2114	14.3	90	70	Short pulse d2, autotomy
Ap2109	13.5	95	80	Short pulse d2, autotomy
Ap2108	13.2	95	70	Short pulse d2, autotomy
Ap2101	15.5	95	70	Short pulse d12,

				original tail
Ap2104	13.0	90	70	Short pulse d12, original tail
Ap2110	14.9	90	70	Short pulse d12, original tail
Ap2124	14.1	95	80	Short pulse d32, original tail
Ap2119	13.0	85	70	Short pulse d32, original tail
Ap2117	15.8	100	72	Short pulse d32, original tail
Ap2129	13.9	90	70	Long pulse d7, original tail
Ap2130	14.0	90	75	Long pulse d7, original tail
Ap2112	15.7	90	75	Long pulse d7, original tail
Ap2123	14.1	90	65	Long pulse 140d chase, original tail
Ap2125	15.9	90	68	Long pulse 140d chase, original tail
Ap2128	13.6	85	75	Long pulse 140d chase, original tail

Table S2. Short pulse/no chase leopard gecko data

Gecko	***Mass (g) at start***	***Mass after tail loss***	***Mass at collection***	***Experimental Group***
Ap2126	14	n/a	13.9	Short pulse d2, original tail
Ap2107	15.6	n/a	16.5	Short pulse d2, original tail

Ap2113	12.1	90	12.7	Short pulse d2, original tail
Ap2114	14.3	11.5	13.1	Short pulse d2, autotomy
Ap2109	13.5	11	11.1	Short pulse d2, autotomy
Ap2108	13.2	11.9	*10.3	Short pulse d2, autotomy

*weight loss during experimental period but otherwise normal (bright, alert, responsive, good muscle tone)

Table S3. Short pulse/10-day chase leopard gecko data

Gecko	***Mass (g) at start***	***Mass after tail loss***	***Mass at collection***	***Experimental Group***
Ap2101	15.5	n/a	16.8	Short pulse d12, original tail
Ap2104	13.0	n/a	15.2	Short pulse d12, original tail
Ap2110	14.9	n/a	16.2	Short pulse d12, original tail

Table S4. Short pulse/30-day chase leopard gecko data

Gecko	***Mass (g) at start***	***Mass after tail loss***	***Mass at collection***	***Experimental Group***
Ap2124	14.1	n/a	18.5	Short pulse d32, original tail
Ap2119	13.0	n/a	17.5	Short pulse d32, original tail
Ap2117	15.8	n/a	20.0	Short pulse d32, original tail

Table S5. Cell counts comparing 5-bromo-2'-deoxyuridine (BrdU) incorporation within the ventricular zone of the medial cortex among original-tailed and tail-autotomized leopard geckos.

GROUP	GECKO ID	SUBAREA#	SECTION#	SIDE	DAPI	BRDU
Og	Ap2107	1	85	L	228	13
Og	Ap2107	1	85	R	217	12
Og	Ap2107	1	86	L	294	10
Og	Ap2107	1	86	R	167	6
Og	Ap2107	1	92	L	230	7
Og	Ap2107	1	92	R	187	3
Og	Ap2107	1	94	L	179	4
Og	Ap2107	1	94	R	156	8
Og	Ap2107	2	101	L	206	5
Og	Ap2107	2	101	R	166	1
Og	Ap2107	2	110	L	171	2
Og	Ap2107	2	110	R	124	4
Og	Ap2107	2	112	L	174	9
Og	Ap2107	2	112	R	170	1
Og	Ap2107	3	124	L	180	5
Og	Ap2107	3	124	R	156	2
Og	Ap2107	3	126	L	167	5
Og	Ap2107	3	126	R	192	2
Og	Ap2107	3	130	L	191	4
Og	Ap2107	3	120	R	197	3
Og	Ap2107	4	143	L	165	4
Og	Ap2107	4	143	R	174	2
Og	Ap2107	4	148	L	225	2
Og	Ap2107	4	148	R	201	4
Og	Ap2107	4	149	L	230	1
Og	Ap2107	4	129	R	175	2
AU	Ap2108	1	36	L	260	7
AU	Ap2108	1	36	R	155	8
AU	Ap2108	1	39	L	160	4
AU	Ap2108	1	39	R	191	6
AU	Ap2108	1	42	L	204	6
AU	Ap2108	1	42	R	224	11
AU	Ap2108	2	56	L	214	4
AU	Ap2108	2	56	R	191	5

AU	Ap2108	2	58	L	208	2
AU	Ap2108	2	58	R	111	1
AU	Ap2108	2	62	L	273	5
AU	Ap2108	2	62	R	200	3
AU	Ap2108	3	76	L	164	2
AU	Ap2108	3	79	L	257	3
AU	Ap2108	3	79	R	219	1
AU	Ap2108	3	91	L	177	1
AU	Ap2108	3	91	R	175	4
AU	Ap2108	4	96	L	203	5
AU	Ap2108	4	96	R	255	3
AU	Ap2108	4	99	L	251	8
AU	Ap2108	4	99	R	182	7
AU	Ap2108	4	105	L	245	3
AU	Ap2108	4	105	R	225	3
AU	Ap2109	1	33	L	224	21
AU	Ap2109	1	33	R	222	16
AU	Ap2109	1	35	R	189	11
AU	Ap2109	1	39	L	232	12
AU	Ap2109	1	39	R	231	19
AU	Ap2109	2	44	L	238	17
AU	Ap2109	2	44	R	213	11
AU	Ap2109	2	47	L	232	2
AU	Ap2109	2	47	R	232	6
AU	Ap2109	2	51	L	234	2
AU	Ap2109	2	51	R	263	12
AU	Ap2109	3	56	L	237	4
AU	Ap2109	3	56	R	249	7
AU	Ap2109	3	58	L	192	3
AU	Ap2109	3	58	R	105	8
AU	Ap2109	3	61	L	222	4
AU	Ap2109	3	61	R	164	1
AU	Ap2109	4	71	L	176	4
AU	Ap2109	4	71	R	182	9
AU	Ap2109	4	76	L	215	10
AU	Ap2109	4	76	R	234	7
AU	Ap2109	4	77	L	199	7
AU	Ap2109	4	77	R	224	15
Og	Ap2113	1	59	L	211	11

Og	Ap2113	1	59	R	241	17
Og	Ap2113	1	62	L	220	7
Og	Ap2113	1	62	R	146	3
Og	Ap2113	1	66	L	104	8
Og	Ap2113	1	66	R	245	16
Og	Ap2113	2	73	L	147	3
Og	Ap2113	2	73	R	180	10
Og	Ap2113	2	76	L	172	9
Og	Ap2113	2	76	R	156	7
Og	Ap2113	2	79	L	203	10
Og	Ap2113	2	79	R	155	2
Og	Ap2113	3	86	L	233	12
Og	Ap2113	3	86	R	211	7
Og	Ap2113	3	89	L	184	12
Og	Ap2113	3	89	R	189	10
Og	Ap2113	3	92	L	238	10
Og	Ap2113	3	92	R	184	1
Og	Ap2113	4	104	L	243	10
Og	Ap2113	4	104	R	219	9
Og	Ap2113	4	107	L	260	12
Og	Ap2113	4	107	R	127	2
Og	Ap2113	4	110	L	264	14
Og	Ap2113	4	110	R	139	5
AU	Ap2114	1	19	L	227	29
AU	Ap2114	1	19	R	180	19
AU	Ap2114	1	22	L	251	20
AU	Ap2114	1	22	R	205	26
AU	Ap2114	1	25	L	100	25
AU	Ap2114	1	25	R	170	22
AU	Ap2114	2	33	L	203	21
AU	Ap2114	2	36	L	177	28
AU	Ap2114	2	36	R	225	24
AU	Ap2114	2	39	L	224	6
AU	Ap2114	2	39	R	196	18
AU	Ap2114	3	46	L	194	13
AU	Ap2114	3	46	R	191	13
AU	Ap2114	3	49	L	185	6
AU	Ap2114	3	49	L	190	14
AU	Ap2114	3	52	R	215	18

AU	Ap2114	4	62	L	194	17
AU	Ap2114	4	62	R	201	22
AU	Ap2114	4	64	L	265	20
AU	Ap2114	4	64	R	217	10
AU	Ap2114	4	67	L	222	15
AU	Ap2114	4	67	R	203	6
Og	Ap2126	1	168	L	187	3
Og	Ap2126	1	168	R	196	5
Og	Ap2126	1	171	L	249	14
Og	Ap2126	1	171	R	186	10
Og	Ap2126	1	174	L	206	9
Og	Ap2126	1	174	R	166	14
Og	Ap2126	2	148	R	183	3
Og	Ap2126	2	151	L	124	8
Og	Ap2126	2	151	R	149	5
Og	Ap2126	2	154	L	204	10
Og	Ap2126	2	154	R	199	15
Og	Ap2126	3	128	L	190	0
Og	Ap2126	3	128	R	173	3
Og	Ap2126	3	132	L	200	1
Og	Ap2126	3	132	R	190	4
Og	Ap2126	3	134	L	159	5
Og	Ap2126	3	134	R	191	4
Og	Ap2126	4	106	L	155	6
Og	Ap2126	4	106	L	198	3
Og	Ap2126	4	109	L	145	0
Og	Ap2126	4	109	R	174	3
Og	Ap2126	4	112	R	169	6

Au, autotomized tail; DAPI, 4’,6-diamindino-2-pheylindole; L, left medial cortex; Og, original tail, R, right medial cortex

Table S6. Long pulse/no chase leopard gecko data

Gecko	***Mass (g) at start***	***Mass after tail loss***	***Mass at collection***	***Experimental Group***
Ap2129	13.9	n/a	15.3 (male)	Long pulse d7, original tail
Ap2130	14.0	n/a	15.2 (female)	Long pulse d7, original tail
Ap2112	15.7	n/a	16.6 (male)	Long pulse d7, original tail

Table S7. Long pulse/20 week chase leopard gecko data

Gecko	***Mass (g) at start***	***Mass after tail loss***	***Mass (g) at collection***	***Experimental Group***
Ap2123	14.1	n/a	26.5	Long pulse 140d chase, original tail
Ap2125	15.9	n/a	Deceased Oct.4	Long pulse 140d chase, original tail
Ap2128	13.6	n/a	25.5	Long pulse 140d chase, original tail
Ap2106*	24.8	n/a	32.1	Long pulse 140d chase, original tail

* Replacement for Ap21-25, collected Mar. 16, 2017

File S1

Hematoxylin and Eosin Protocol

Slide-mounted sections were deparaffinized and rehydrated to deonized to water through xylene (3 washes; 2 minutes), absolute isopropanol (3 washes; 2 minutes), 70% isopropanol (2 minutes) and deionized water (dH_2O) (2 minutes). Sections were stained with modified Harris hematoxylin for 10 minutes (Fischer Scientific, Waltham, Massachusetts, USA), and then rinsed with deionized water before being dipped (5 times) in acid alcohol (1% hydrochloric acid in 70% isopropanol). Sections were then rinsed again in deionized water, blued in ammonia water (~10 seconds), followed by a rinse in running water. Next, sections were dipped 6 times in 70% isopropanol before being stained in eosin (1 minute). Sections were passed through four changes of 100% isopropanol (2 minutes each), cleared in three changes of xylene (2 minutes each), and then coverslipped using Cytoseal (Fischer Scientific, Waltham, Massachusetts, USA).

Immunofluorescence Protocols

Sections were de-paraffinized and rehydrated to water through xylene (3 washes; 2 minutes), absolute isopropanol (3 washes; 2 minutes), 70% isopropanol (2 minutes) and deionized water (dH2O) (2 minutes). Sections were then rinsed for 15 minutes in 1X phosphate buffered saline (PBS), and subject to one of two antigen retrieval methods: sections stained with SOX2/Musashi-1 or SOX2/HuCD were incubated for 30 minutes in 2N hydrochloric acid at 37 ˚C; all other sections were submerged in citrate buffer at 95˚C for 12 minutes, and then cooled for 20 minutes. Following antigen retrieval, all

sections were rinsed for 2 minutes in 1X PBS. Sections were then incubated for 20 minutes in 0.1% trypsin at 37˚C (Sigma-Aldrich, St. Louis, Missouri, USA), rinsed for 2 minutes in 1X PBS and then blocked for 30 minutes at 37 ˚C in 5% normal goat serum in diluent (1% bovine serum albumin, 0.5% Tween 20, 0.1% sodium azide in 1X PBS). Next, sections were incubated overnight at 4˚C in primary antibody diluted in diluent (SOX2 [1:50] Cell-Signalling, Whitby, Ontario, Canada; GFAP [1:400] DAKO, Glostrup, Denmark; Vimentin [1:50] Developmental Studies Hybridoma Bank, Iowa City, Iowa, USA; HuC/D [1:10] Molecular Probes, Rockford, Illinois, USA; BrdU [1:200] Developmental Studies Hybridoma Bank, Iowa City, Iowa, USA; tomato lectin [1:50] Sigma-Aldrich, St. Louis, Missouri, USA; NeuN [1:125] Abcam, Cambridge, Massachusetts, USA). One section per slide served as an omission control and was incubated in diluent only. Slides were then rinsed in 1X PBS (3 washes; 2 minutes), and incubated in secondary antibody at room temperature for 1 hour. Slides were rinsed in 1X PBS (3 washes; 2 minutes), and then stained with nuclear marker DAPI ([1:5000] Life Technologies, Eugine, Oregon, USA) (2 minutes). Once more, slides were rinsed in 1X PBS (3 washes; 2 minutes), and then cover slipped with fluorescent mounting media (DAKO, Glostrup, Denmark).

To detect PCNA, pHH3, FGF2, VEGF, VEGFR1 and VEGFR2, we used an abbreviated protocol. As for the Standard Protocol, sections were de-paraffinized and brought to water. Sections then underwent heat-induced antigen retrieval by submersion in citrate buffer at 95˚C for 12 minutes, and were subsequently cooled for 20 minutes. Following antigen retrieval sections were rinsed in phosphate buffered saline (PBS). They were then blocked for one hour in 5% normal goat serum (NGS) in

sterile PBS at room temperature. Primary antibodies were diluted in sterile PBS and applied to one section on the slide for overnight incubation at 4°C (pHH3 [1:100] Cell Signaling, Whitby, Ontario, Canada; PCNA [1:100], FGF2 [1:100], VEGF [1:50], VEGFR1 [1:50], VEGFR2 [1:100] Santa-Cruz Biotechnology, Dallas, Texas, USA). Sections were rinsed in PBS (3 washes; 2 minutes) and then incubated in secondary antibody for one hour at room temperature. As for the Standard Protocol, slides were then rinsed with PBS, counterstained using DAPI, rinsed with PBS (3 washes; 2 minutes) and cover slipped using fluorescent mounting media (DAKO, Glostrup, Denmark).

Immunohistochemistry Protocol

Immunohistochemistry was additionally used to visualize pHH3. Sections were de-paraffinized and rehydrated to water as above, quenched for 20 minutes in 3% hydrogen peroxide diluted in deionized water, and rinsed with PBS (3 washes; 2 minutes). Sections underwent heat-induced antigen retrieval by submersion in citrate buffer (see above), prior to rinsing with PBS. They were then blocked for one hour in 3% normal goat serum (NGS) in sterile PBS at room temperature. pHH3 [1:500] was diluted in sterile PBS and applied to one section on the slide for overnight incubation at 4°C. Sections were rinsed in PBS and incubated in secondary antibody for one hour at room temperature (Biotinylated Goat anti-Rabbit [1:500] Jackson Immuno Research Laboratories). Following subsequent PBS rinsing, sections were incubated with horseradish peroxidase conjugated streptavidin (Jackson ImmunoResearch Laboratories, Inc. West Grove, Pennsylvania, USA) diluted in sterile PBS (one hour; room temperature). Sections were rinsed in PBS and then 3,3’-diaminobenzidine

peroxidase substrate (DAB; Vector Laboratories, Burlingame, California, USA) was applied for 35 seconds, before the chromogenic reaction was stopped by immersion in deionized water. Sections were counterstained with Mayer’s hematoxylin (1 minute), rinsed in deionized water, blued in ammonia water, and rinsed again in deionized water. Slides were then dehydrated through three changes of 100% isopropanol (2 minutes each), cleared in three changes of xylene (2 minutes each), and coverslipped using Cytoseal.
